# StreptInCor, a Group A Streptococcal Adsorbed Vaccine: Evaluation of Repeated Intramuscular Dose Toxicity Testing in Rats

**DOI:** 10.3389/fcvm.2021.643317

**Published:** 2021-05-11

**Authors:** Luiz Carlos de Sá-Rocha, Lea Maria Macruz Ferreira Demarchi, Edilberto Postol, Roney Orismar Sampaio, Raquel Elaine de Alencar, Jorge Kalil, Luiza Guilherme

**Affiliations:** ^1^Neuroimmunology Laboratory School of Veterinary Medicine and Animal Sciences, University of São Paulo, São Paulo, Brazil; ^2^Heart Institute (InCor), School of Medicine, University of São Paulo, São Paulo, Brazil; ^3^Immunology Investigation Institute, National Institute for Science and Technology, University of São Paulo, São Paulo, Brazil

**Keywords:** *S. pyogenes*, vaccine, safety, histopathology, toxicity

## Abstract

*Streptococcus pyogenes* infections continue to be a worldwide public health problem, causing various diseases in humans, with rheumatic fever and rheumatic heart disease being the most harmful manifestations. Impetigo and post-streptococcal glomerulonephritis are also important sequelae of skin infections. We have developed a candidate vaccine epitope (StreptInCor) that presents promising results in diverse animal models. To assess whether the StreptInCor alum-adsorbed vaccine could induce undesirable effects, a certified independent company conducted a repeated intramuscular dose toxicity evaluation in Wistar rats, a choice model for toxicity studies. We did not observe significant alterations in clinical, hematological, biochemical, anatomical, or histopathological parameters due to vaccine administration, even when the animals received the highest dose. In conclusion, repeated intramuscular doses did not show signs of macroscopic or other significant changes in the clinical or histopathological parameters, indicating that StreptInCor can be considered a safe candidate vaccine.

## Introduction

*Streptococcus pyogenes*, also known as group A streptococcus (GAS), is a Gram-positive bacterium responsible for some human diseases, such as pharyngitis, impetigo, and post-streptococcal complications, including glomerulonephritis and rheumatic fever (RF) and severe sequelae of rheumatic heart disease (RHD). The highest incidence of infections by GAS occurs in children between 5 and 15 years of age, especially in children living in poor sanitation conditions (1). Bowen et al. performed a systematic review and estimated that more than 162 million children younger than 15 years had impetigo and the broader condition pyoderma in low-income and low-middle-income countries (2). A meta-analysis study conducted by Shaikh et al. estimated that the prevalence of GAS infection among children of all ages who present with sore throat was 37%, and in those younger than 5 years, it was 24%. The prevalence of GAS carriage among well children with no signs or symptoms of pharyngitis was 12% (3). A recent study reported the occurrence of ~319,000 deaths from RHD-related complications. Although the health-related burden of RHD has declined worldwide, high disease rates persist in the world's poorest regions (1).

RF generally develops after the occurrence of untreated streptococcal pharyngitis and affects ~0.3–3% of susceptible individuals. Among the manifestations of RF, RHD is the most important sequelae and affects 45% of RF patients, and it is capable of causing serious damage to the aortic and mitral valves, leading to cardiac dysfunction (4). In Brazil, RHD accounts for 90% of cardiac surgeries in children (5).

Although not fully elucidated, RF/RHD develops from the involvement of autoimmune mechanisms mediated by molecular mimicry in genetically predisposed individuals. Genetic susceptibility is related to several gene polymorphisms of placebo innate and adaptive immune responses (6–8).

In the case of RHD, the activation of T and B lymphocytes may lead to the production of antibodies and T lymphocytes reactive against some epitopes of the N-terminal region of the M protein, which may react against myocardial and valvar tissue proteins due to their similarity to pathogen epitopes (9–13).

GAS strains are currently identified by sequencing the hypervariable N-terminal region of the *emm* gene encoding the M protein, and they have already been classified into more than 220 *emm* types, with some identified as rheumatogenic strains (14–16). These findings suggest that not all strains contain the epitopes capable of triggering RF/RHD but are probably involved with other manifestations, such as impetigo or glomerulonephritis.

Since the pioneering studies conducted by Rebecca Lancefield in 1962, the M protein has been the target of choice for a streptococcal vaccine (17). The M protein is formed by two coil-coiled chains with a flexible structure located on the bacterial wall and represents an important virulence factor that is highly immunogenic. Unlike the polymorphic N-terminal region of the M protein, the C-terminal region is highly conserved among the different serotypes (15, 18).

Vaccines based on both the N- and C-terminal regions of the M protein have been proposed in the last 14 years using new technologies, such as recombinant proteins or synthetic peptides (19–22).

The present work focused on StreptInCor, a candidate vaccine that contains 55 synthetic amino acid residues of the C-terminal region of the M protein. The formulation of StreptInCor with aluminum hydroxide (alum) was tested in inbred, outbred, and transgenic mice harboring human HLA class II alleles. High titers of specific antibodies and no cross-reactions with cardiac proteins or deleterious effects in vaccinated animals were observed (23–25).

The aim of the present toxicology work was to verify whether the candidate vaccine triggers toxic effects and identify target organs and deleterious effects on animal physiology and hematological, biochemical, anatomical, and histopathological parameters.

## Materials and Methods

Repeated intramuscular (IM) dose toxicity tests under conditions of good laboratory practice (GLP) and following national (26) and international standards were conducted by TECAM - Tecnologia Ambiental (São Roque, SP, Brazil), and they were identified as 5517/2015IM, protocol 27 (27, 28). All procedures were in accordance with national guidelines of the National Animal Experiment Placebo Council (CONCEA) and Committee for Animal Care and Use (COBEA) and international requirements based on the “Guide for the Care and Use of Laboratory Animals” (29).

The Wistar rats were kept in a facility accredited by the Association for Assessment and Accreditation of Laboratory Animal Care (AAALAC) and Credenciamento Institucional para Atividades com Animais em Ensino ou Pesquisa (CIAEP)/CONCEA under number 01.0242.214.

**Figure 1:**
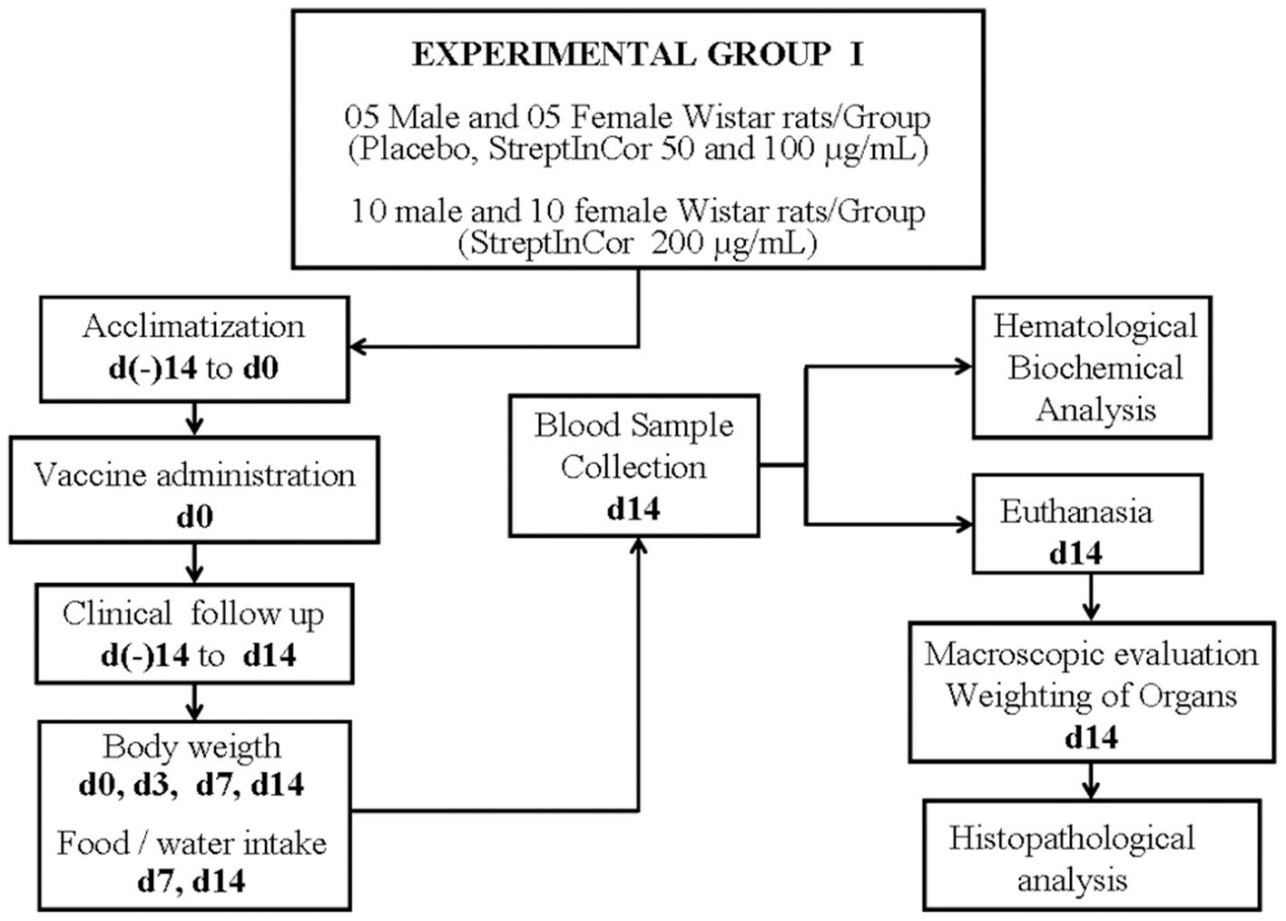
Experimental design.

**Figure 2:**
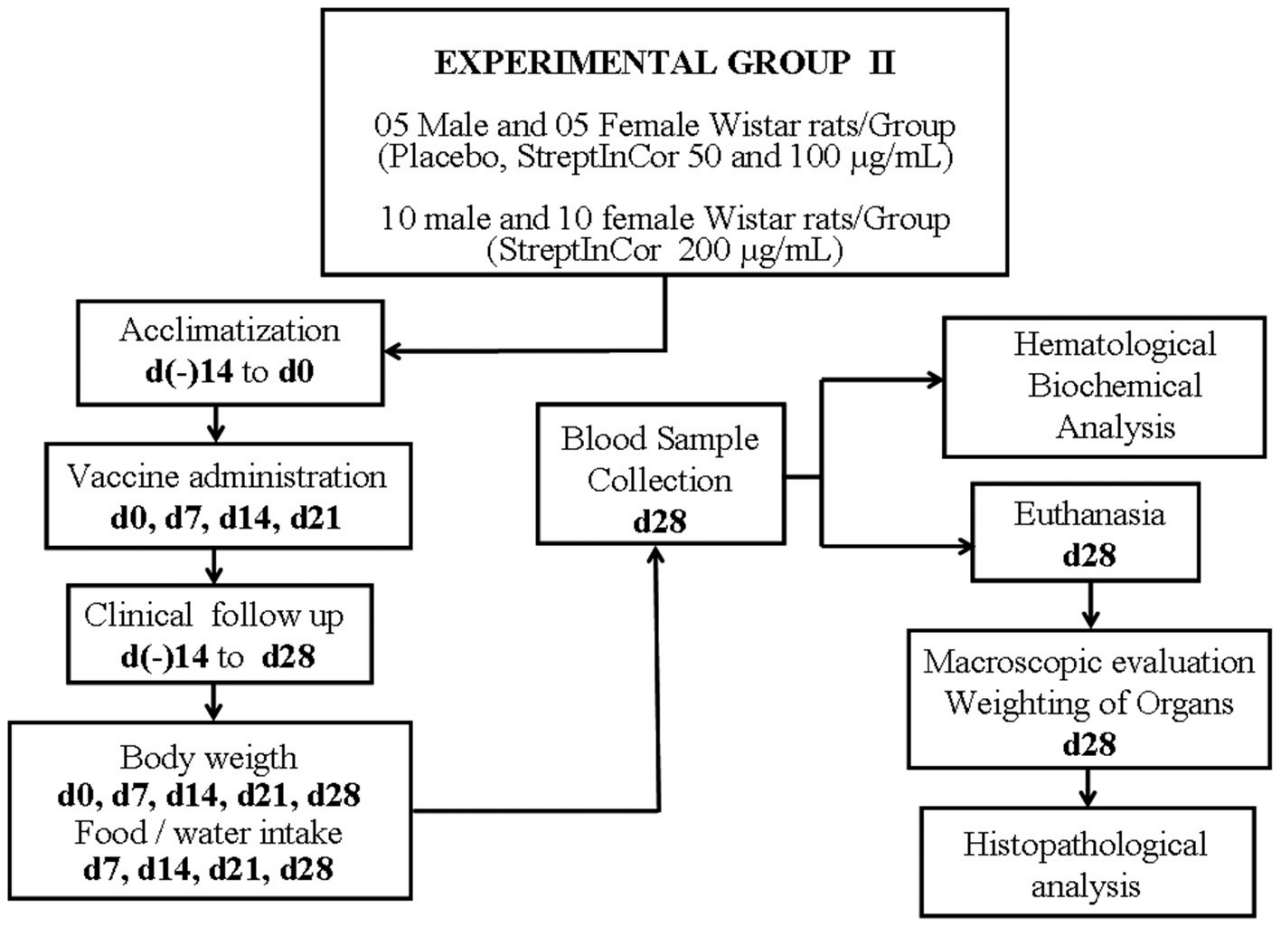
Experimental design.

The experimental design of the rat IM toxicity tests is shown in Figures 1, 2.

### StreptInCor Candidate Vaccine

PolyPeptide Laboratories Inc. (Torrance, CA, USA) manufactured the synthetic peptide StreptInCor based on good manufacturing practice (GMP) standards. The vaccine formulation was prepared at Butantan Institute (São Paulo, Brazil). StreptInCor in different doses are emulsified in aluminum hydroxide as adjuvant, as follows: lot 15055 (50 μg/ml, low dose); lot 15056 (100 μg/ml, medium dose); lot 15057 (200 μg/ml, high dose); and lot 15054 (placebo formulation).

### Animals

Outbred male and female Wistar rats (*Rattus norvegicus*) aged 7–9 weeks were bred at TECAM - Tecnologia Ambiental.

Both acute IM toxicity tests and repeated IM dose toxicity tests were evaluated. Only rats deemed healthy after evaluation by a veterinarian and without any clinical manifestation were selected for the study. The animals were acclimated 5 days before the studies and kept in monitored rooms at average temperature and humidity conditions of 20.8°C and 71.2%, respectively. The rats received species-specific rations (Nuvilab CR-1, Quimtia S.A., Colombo, PR, Brazil) and filtered water *ad libitum*. They were maintained in a 12-h light/dark cycle with ~10–12 air changes per hour.

### Administration of the StreptInCor Candidate Vaccine

The candidate vaccine was formulated at the concentrations of 50, 100, and 200 μg/ml of StreptInCor, plus alum (0.5 mg/ml of aluminum hydroxide), thimerosal (0.004 mg/ml), and phosphate buffered saline solution.

Previously identified animals received one IM injection of 250 μl of StreptInCor or placebo solution on the thigh muscles of their hind limbs, which had been trichotomized.

The toxicity due to acute immune response was evaluated in Experimental Group I, which included 50 Wistar rats (25 males and 25 females). On day 0, the rats received only one IM injection of 250 μl of StreptInCor with different concentrations, as follows: 50 μg/ml (low dose) (five males and five females); 100 μg/ml (medium dose) (five males and five females); and 200 μg/ml (high dose) (10 males and 10 females). Animals of placebo subgroup (five males and five females) received only one IM injection of 250 μl of a saline plus aluminum hydroxide solution, on day 0.

The toxicity effect of StreptInCor repeated doses was evaluated in Experimental Group II, which included 50 Wistar rats (25 males and 25 females). On days 0, 7, 14, and 21, the animals received one IM injection of 250 μl of StreptInCor with different concentrations, as follows: 50 μg/ml (low dose) (five males and five females); 100 μg/ml (medium dose) (five males and five females); and 200 μg/ml (high dose) (10 males and 10 females). Rats of placebo subgroup (five males and five females) received one IM injection of 250 μl of a saline plus aluminum hydroxide solution, on days 0, 7, 14, and 21.

A recovery group of rats underwent the same protocol of the Experimental Group II protocol and recovered for 2 weeks before euthanasia on day 42. They underwent two additional measurements for body weight and food consumption, on days 35 and 42.

### Clinical Follow-Up

The animals were individually monitored throughout the acclimation as well as during the experimental period. Experienced veterinarians classified the severity of clinical signs as mild, moderate, or severe based on physical examination. Clinical observations included changes in the fur, skin, eyes, and mucous membranes; occurrence of secretions and excretions; and autonomic activity (lacrimation, piloerection, changes in the pupils, and respiratory pattern). The veterinarians were also vigilant for possible changes in gait, posture, and reaction to manipulation as well as the presence of tonic or clonic movements and stereotypies, such as excessive grooming, repetitive circulatory movements, self-mutilation, and walking backwards. Finally, local tolerance was assessed by the presence of edema, erythema, desquamation, wounding, alopecia, and any other signs of local irritation and/or inflammation.

The body weights of the animals were measured on days 0, 3, 7, and 14 for Group I and on days 0, 7, 14, 21, and 28 for Group II. Food consumption was measured on days 7 and 14 for Group I and on days 7, 14, 21, and 28 for Group II. The recovery group had two additional measurements for body weights and food consumption (days 35 and 42).

### Euthanasia

All animals were sedated by an IM injection of a combination of xylazine (8 mg/kg) and ketamine (65 mg/kg). Blood samples were collected for hematological and biochemical analyses through cardiac puncture after deep anesthesia and prior to euthanasia.

The veterinarians euthanized the rats by exsanguination after confirmation of deep anesthesia. After 2 weeks (day 14) and 4 weeks (day 28), rats from Groups I and II were euthanized, respectively. Rats from recovery group were euthanized after 6 weeks, on day 42.

### Hematological and Biochemical Analyses

Hematological tests, including the red blood cell count, hemoglobin concentration, hematocrit, mean corpuscular volume (MCV), mean corpuscular hemoglobin (MCH), MCH concentration (MCHC), white blood cell count, and platelet count were evaluated using HEMATOClin 2.8 Vet (QUIBASA Bioclin, Brazil).

Commercial kits for biochemical parameters were used to determine the serum levels of glucose, total cholesterol, urea, creatinine, alanine aminotransferase, aspartate aminotransferase, albumin, globulin, total protein, sodium, chloride, and potassium. The results were obtained by spectrometry in liquid medium by using the semiautomatic TP Analyzer (Thermo-Plate, Brazil).

### Necropsy, Wet Organ Weight, and Histopathological Analysis

Necropsies of all the animals were performed by veterinarians who collected tissue samples for histological analysis. Organs such as the brain, liver, kidneys, adrenal glands, heart, gonads (testis and epididymis or ovary), thymus, and spleen were collected; and their relative organ weight (weight of organ as a percentage of the total body weight of each rat) was calculated and compared with the relative organ weight of the placebo group rats. After macroscopic evaluation, tissue samples of the brain, lung, stomach, esophagus, small intestine, large intestine, liver, pancreas, kidney, adrenal glands, heart, gonad (testis or ovary), urinary bladder, femur, thymus, spleen, and lymph nodes were fixed in 10% buffered formalin. Fixed samples were dehydrated in alcohol baths in a concentration gradient, cleared in xylene, and embedded in paraffin blocks. Five micro sections were cut and mounted on slides and stained by the hematoxylin and eosin method.

### Statistical Analysis

For each parameter analyzed, the data are expressed as the mean ± the standard error of the mean (SEM) between the different animals of each group. All data were examined by the D'Agostino and Pearson omnibus normality tests to verify whether the data could be analyzed by parametric tests. When the data passed the normality test, we employed a one-way analysis of variance and Tukey's post-test to compare all pairs of columns. If the data did not pass the normality test, we used the Kruskal–Wallis test and Dunn's post-test to compare all pairs of columns. We used GraphPad Prism software version 5.01 for Windows (GraphPad Software, San Diego California USA, www.graphpad.com) to analyze the data, and *p*-values < 0.05 were considered significant.

## Results

Clinical signs of toxicity or intolerance at the site of administration and mortality were not observed during the acute toxicity study. In addition, the animals used in this experiment showed body weight gains from 220 to 300 g during acute phase (Group I) and 180 to 250 g during chronic phase (Group II) consistent with the observed food intake.

Animals from all groups displayed chronic granulomatous inflammation in skeletal muscle at the site of injection: 66.7% of the animals in the placebo (two males and two females); and 66.7% (two males and two females), 16.7% (one female), and 50.0% (two males and one female) of the animals treated with 50, 100, and 200 μg of StreptInCor, respectively. Considering that animals from all groups studied in the acute toxicity test showed good tolerance to StreptInCor, we maintained the same concentrations to evaluate the repeated dose toxicity.

### Clinical Follow-Up

No mortality or clinical signs related to the injection of StreptInCor at 50, 100, or 200 μg/ml were recorded. Food intake and water intake were similar between the placebo and experimental groups. In addition, the weight gains were similar among animals of all groups, being around 220 g in placebo and 150 g in immunized animals.

### Hematological and Biochemical Analyses

The repeated injections at different doses of the candidate vaccine did not interfere markedly with the hematology data; however, we observed that erythrocytes from the male rats that received 50 μg/ml differed from those obtained in the placebo group (*p* < 0.05). On the other hand, females vaccinated with 50 μg/ml presented an increased leukocyte count (*p* < 0.05) than the placebo group due to an increased number of lymphocytes (*p* < 0.05) (Table 1).

**Table 1:** Hematology data from Wistar rats vaccinated with StreptInCor at different doses (50, 100 or 200 μg/mL) compared with placebo.

**Males**	**Placebo**	**50 μg/mL**	**100 μg/mL**	**200 μg/mL**
RBC (10^12^/L)	9.36 ± 0.14	7.78 ± 0.13^*^	8.34 ± 0.12	8.26 ± 0.20
Hemoglobin (g/L)	172.60 ± 6.33	145.80 ± 2.52	150.60 ± 2.77	154.50 ± 4.33
Hematocrit (fraction)	0.52 ± 0.02	0.44 ± 0.01	0.46 ± 0.01	0.45 ± 0.01
MCV (fL)	55.29 ± 1.51	56.40 ± 1.02	54.92 ± 1.06	54.89 ± 0.73
MCH (pg)	18.43 ± 0.50	18.76 ± 0.28	18.07 ± 0.45	18.70 ± 0.18
MCHC (g/L)	333.30 ± 0.21	332.70 ± 2.04	329.00 ± 2.23	340.80 ± 2.33
Platelets (10^9^/L)	910.80 ± 116.40	913.50 ± 51.40	856.20 ± 85.00	773.90 ± 123.00
Leukocytes (10^9^/L)	6.03 ± 0.75	5.50 ± 0.35	7.46 ± 0.76	5.36 ± 0.42
Lymphocytes(10^9^/L)	4.97 ± 0.63	4.52 ± 0.32	6.14 ± 0.67	4.26 ± 0.33
Monocytes (10^9^/L)	0.13 ± 0.01	0.12 ± 0.01	0.13 ± 0.01	0.10 ± 0.01
Granulocytes (10^9^/L)	0.94 ± 0.16	0.86 ± 0.05	1.19 ± 0.15	1.01 ± 0.11
Lymphocytes (fraction)	0.82 ± 0.02	0.82 ± 0.01	0.82 ± 0.02	0.80 ± 0.02
Monocytes (fraction)	0.02 ± 0.00	0.02 ± 0.00	0.02 ± 0.00	0.02 ± 0.00
Granulocytes (fraction)	0.15 ± 0.02	0.16 ± 0.01	0.16 ± 0.02	0.19 ± 0.02
**Females**	**Placebo**	**50 μg/mL**	**100 μg/mL**	**200 μg/mL**
RBC (10^12^/L)	7.65 ± 0.01	7.77 ± 0.26	7.53 ± 0.19	7.78 ± 0.19
Hemoglobin (g/L)	141.20 ± 1.24	142.00 ± 3.05	136.60 ± 2.98	144.00 ± 2.70
Hematocrit (fraction)	0.42 ± 0.01	0.43 ± 0.01	0.41 ± 0.01	0.43 ± 0.01
MCV (fL)	54.54 ± 0.18	55.24 ± 0.72	54.99 ± 0.38	55.10 ± 0.58
MCH (pg)	18.46 ± 0.13	18.29 ± 0.27	18.15 ± 0.11	18.54 ± 0, 21
MCHC (g/L)	338.50 ± 1.88	331.20 ± 1.27	330.10 ± 1.65	336.60 ± 1.78
Platelets (10^9^/L)	802.00 ± 48.14	947.40 ± 93.40	769.60 ± 52.30	800.80 ± 112.20
Leukocytes (10^9^/L)	4.08 ± 0.37	7.40 ± 0.47^*^	2.44 ± 0.38	4.15 ± 0.30
Lymphocytes (10^9^/L)	3.16 ± 0.30	6.00 ± 0.40^*^	1.78 ± 0.34	3.41 ± 0.29
Monocytes (10^9^/L)	0.09 ± 0.01	0.13 ± 0.01	0.06 ± 0.00	0.06 ± 0.00
Granulocytes (10^9^/L)	0.84 ± 0.17	1.26 ± 0.07	0.60 ± 0.06	0.68 ± 0.03
Lymphocytes (fraction)	0.77 ± 0.03	0.81 ± 0.05	0.71 ± 0.05	0.81 ± 0.01
Monocytes (fraction)	0.02 ± 0.00	0.02 ± 0.00	0.024 ± 0.00	0.02 ± 0.00
Granulocytes (fraction)	0.21 ± 0.03	0.17 ± 0.00	0.27 ± 0.05	0.17 ± 0.01

*Each value represents the mean ± SEM of placebo, StreptInCor at 50, 100 and 200 μg/mL. RBC, red blood cells; MCV, mean cell volume; MCH, mean cell hemoglobin; MCHC, mean cell hemoglobin concentration. Dunn's Multiple Comparison test:*

* *significant differences: males- StreptInCor (50 μg/mL) and placebo (p < 0.05) and leukocytes and lymphocytes from females treated with StreptInCor (50 μg/mL) and placebo (p < 0.05)*.

Metabolic alterations due to the injection of placebo or the different doses of StreptInCor candidate vaccine were not observed (Table 2).

**Table 2:** Biochemistry data from Wistar rats vaccinated with repeated injections of StreptInCor at 50, 100 or 200 μg/mL doses compared with placebo.

**Males**	**Placebo**	**50 μg/mL**	**100 μg/mL**	**200 μg/mL**
Urea (mmol/L)	16.94 ± 1.91	15.60 ± 0.46	21.49 ± 1.15	16.26 ± 0.86
Creatinine (μmol/L)	42.43 ± 3.92	29.88 ± 8.64	57.11 ± 16.32	37.66 ± 3.18
AST (IU/L)	125.20 ± 15.18	78.40 ± 18.79	115.90 ± 12.64	115.40 ± 14.50
ALT (IU/L)	30.46 ± 8.72	27.94 ± 5.48	34.78 ± 1.72	32.39 ± 2.67
Glucose (mmol/L)	9.94 ± 1.63	12.57 ± 0.77	12.39 ± 1.34	12.43 ± 0.57
Cholesterol (mmol/L)	2.24 ± 0.42	1.85 ± 0.11	1.78 ± 0.05	1.75 ± 0.10
Albumin (g/L)	31.30 ± 0.86	34.46 ± 1.59	29.76 ± 1.11	29.09 ± 0.57
Globulin (g/L)	38.06 ± 3.81	41.08 ± 4.18	43.22 ± 5.39	35.66 ± 1.46
Total Protein (g/L)	69.36 ± 4.67	75.54 ± 5.58	72.98 ± 6.48	64.75 ± 1.97
**Females**	**Placebo**	**50 μg/mL**	**100 μg/mL**	**200 μg/mL**
Urea (mmol/L)	21.32 ± 1.40	16.72 ± 1.15	13.94 ± 1.51	15.79 ± 1.08
Creatinine (μmol/L)	47.38 ± 8.03	56.22 ± 2.45	56.40 ± 5.67	55.13 ± 3, 0
AST (IU/L)	125.60 ± 16.28	125, 60 ± 25.53	109.20 ± 6.15	81.28 ± 5.04
ALT (IU/L)	26.00 ± 4.91	31.52 ± 3.32	23.76 ± 4.96	30.71 ± 1.45
Glucose (mmol/L)	9.41 ± 0.55	10.73 ± 1.09	10.07 ± 0.67	11.19 ± 0.58
Cholesterol (mmol/L)	1.68 ± 0.09	1.74 ± 0.13	1.38 ± 0.15	1.83 ± 0.10
Albumin (g/L)	33.20 ± 1.32	40.44 ± 8.62	31.30 ± 0.91	33.27 ± 0.82
Globulin (g/L)	33.92 ± 2.62	30.46 ± 1.86	30.94 ± 1.37	32.27 ± 0.82
Total Protein (g/L)	67.12 ± 3.88	70.90 ± 8.68	62.24 ± 2.07	65.54 ± 1.47

*Each value represents the mean ± SEM of placebo, StreptInCor at 50, 100, and 200 μg/mL. AST, aspartate aminotransferase; ALT, alanine aminotransferase. Dunn's Multiple Comparison test, no statistically significant differences were observed*.

### Necropsy, Wet Organ Weight, and Histopathological Analysis

No macroscopic alterations were observed. The organ wet weights as well as the relative weights were similar among the experimental groups and placebo animals (Table 3).

**Table 3:** Relative organ weights from the male and female Wistar rats after administration of StreptInCor at 50, 100, 200 μg/mL doses or placebo.

**Males**	**Placebo**	**50 μg/mL**	**100 μg/mL**	**200 μg/mL**
Heart	0.33 ± 0.02	0.30 ± 0.01	0.30 ± 0.01	0.31 ± 0.02
Liver	3.36 ± 0.40	3.70 ± 0.14	3.85 ± 0.15	3.75 ± 0.93
Kidney	0.71 ± 0.05	0.67 ± 0.03	0.69 ± 0.03	0.71 ± 0.02
Adrenal	0.01 ± 0.00	0.02 ± 0.00	0.02 ± 0.00	0.02 ± 0.00
CNS	0.64 ± 0.07	0.57 ± 0.02	0.58 ± 0.01	0.58 ± 0.03
Spleen	0.20 ± 0.02	0.24 ± 0.01	0.20 ± 0.01	0.21 ± 0.01
Thymus	0.16 ± 0.01	0.20 ± 0.02	0.18 ± 0.01	0.16 ± 0.01
Epydidimis+Testicle	1.24 ± 0.08	1.57 ± 0.22	1.60 ± 0.15	1.58 ± 0.09
**Females**	**Placebo**	**50 μg/mL**	**100 μg/mL**	**200 μg/mL**
Heart	0.32 ± 0.01	0.35 ± 0.03	0.33 ± 0.01	0.32 ± 0.01
Liver	3.64 ± 0.16	3.82 ± 0.13	3.48 ± 0.13	3.47 ± 0.13
Kidney	0.76 ± 0.01	0.75 ± 0.02	0.76 ± 0.04	0.78 ± 0.02
Adrenal	0.03 ± 0.01	0.03 ± 0.01	0.048 ± 0.01	0.36 ± 0.00
CNS	0.85 ± 0.02	0.79 ± 0.03	0.86 ± 0.13	0.84 ± 0.02
Spleen	0.23 ± 0.01	0.23 ± 0.01	0.23 ± 0.01	0.25 ± 0.01
Thymus	0.19 ± 0.03	0.22 ± 0.02	0.24 ± 0.00	0.21 ± 0.01
Ovary	0.08 ± 0.01	0.07 ± 0.01	0.10 ± 0.03	0.07 ± 0.01

*Each value represents the mean ± SEM between rats immunized with StreptInCor 50, 100, and 200 μg/mL and placebo. No statistically significant differences were observed by Dunn's Multiple Comparison test*.

The main histological finding observed in all experimental groups was chronic granulomatous inflammation in the skeletal muscle at the site of injection in both the placebo and immunized animals, as follows: 70% of rats in the placebo group (four males and three females); and 70% (four males and three females), 40% (one male and three females), and 65% (six males and seven females) of animals that received 50, 100, and 200 μg of StreptInCor, respectively.

**Figure 3:**
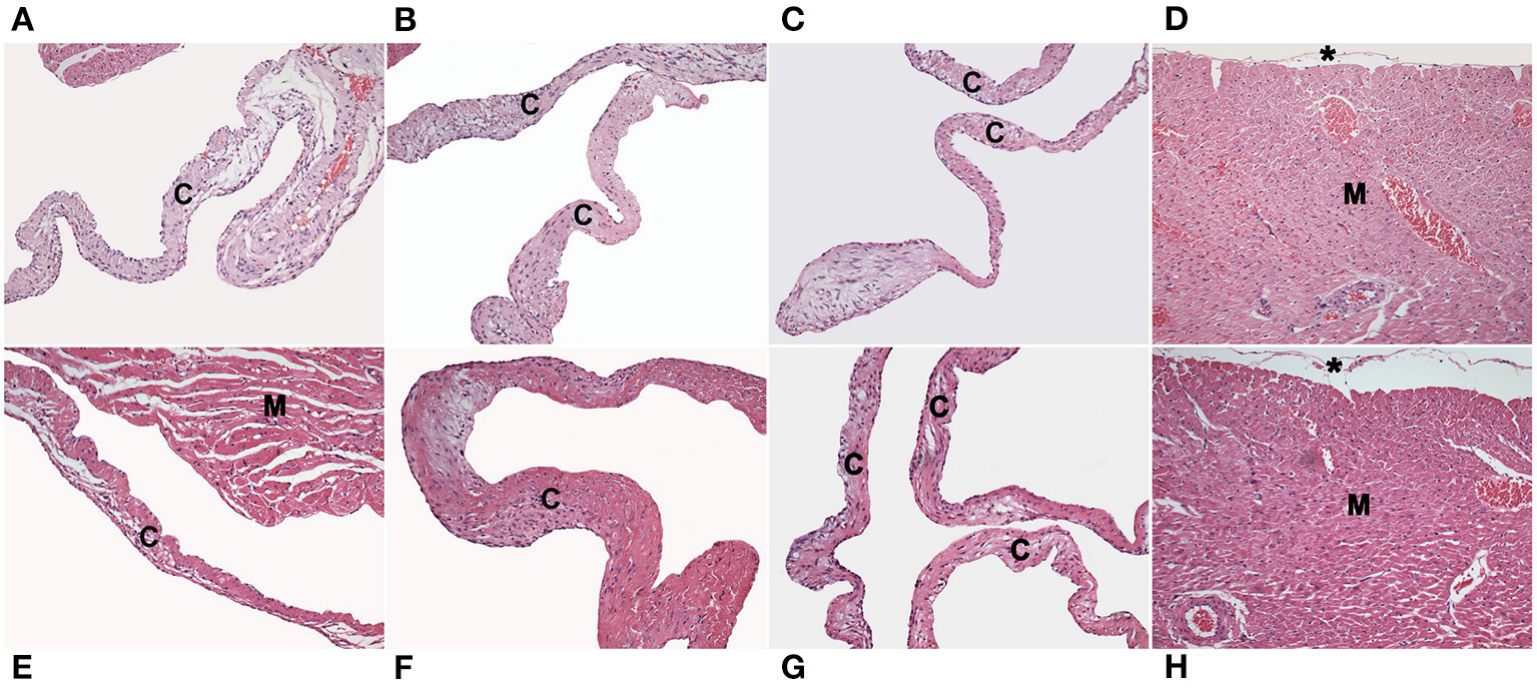
Photomicrographs of histological sections of the tricuspid **(A)**, mitral **(B)** and aortic **(C)** valves, and myocardium **(D)** from Wistar rats injected with placebo. The tricuspid **(E)**, mitral **(F)** and aortic **(G)** valves, and myocardium **(H)** from rats injected with a high dose of StreptInCor (200 μg/ml). There is no necrosis, inflammatory infiltrate, neovascularization, or fibrosis in the valve cusps (c), myocardium (M), or epicardium (*). Hematoxylin and eosin staining, ×200.

**Figure 4:**
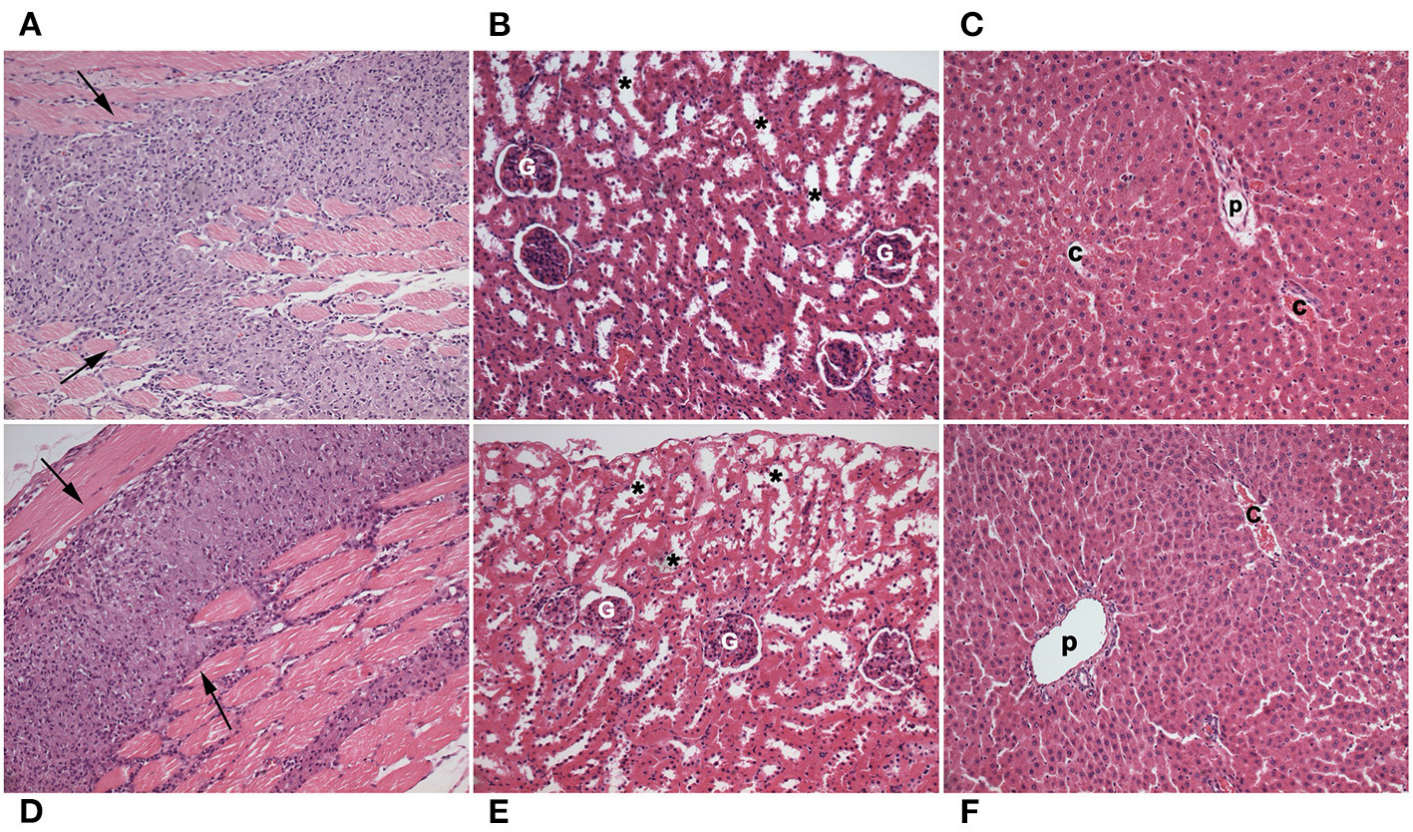
Photomicrographs of histological sections of skeletal muscle **(A)**, kidney **(B)**, and liver **(C)** from Wistar rats injected with placebo, and of skeletal muscle **(D)**, kidney **(E)**, and liver **(F)** from rats injected with high dose of StreptInCor (200 μg/mL). Chronic granulomatous inflammation (arrows) in skeletal muscle at the site of placebo **(A)** and StrepInCor **(D)** injections. There are not necrosis, inflammatory infiltrate, neovascularization, or fibrosis in renal or hepatic tissues. Hematoxylin and eosin staining, ×200. **(G)** Glomerulus; (*) proximal tubules; (p) portal tract; (c) centrilobular hepatic vein.

Histological analysis of the other tissues did not show alterations compared with that of placebo animals, and this response was dependent of the StreptInCor dose (Figures 3, 4).

## Discussion

The present work is part of a set of experiments performed to develop an effective and safe GAS vaccine capable of inducing protection against oropharyngeal infections, RF/RHD, necrotizing fasciitis, and toxic shock syndrome. *Streptococcus pyogenes* is a strictly human pathogen and normally does not induce disease in animals. This fact causes some difficulties to develop an ideal animal model. Despite of it, encouraging results from studies with mice (Swiss, Balb-c, and HLA class II Tg mice) (23–25) and minipigs (30) showed that StreptInCor candidate vaccine is safe.

In this study, we conducted non-clinical safety tests to determine whether StreptInCor candidate vaccine could induce adverse reactions in other animal species.

Because no adverse effects or mortality were observed at any of the concentrations of StreptInCor (50, 100, and 200 μg/ml) in the acute toxicity study (single dose, 14 days), the same formulations were tested in the repeated dose toxicity studies in two species, non-rodents (30) and rodents (Wistar rats), as described here.

No mortality or major changes related to feeding, hydration, or weight gain were observed among the animals. The most important difference in relation to the placebo group was the increased number of leukocytes (lymphocytes, monocytes, and granulocytes) in the females that received 50 μg of StreptInCor.

Repeated immunizations at intervals of 7 days are indicated for evaluation of toxicity effects. Of note, this protocol is not adequate for the evaluation of humoral response (31, 32).

Macroscopic analysis of tissues and organs showed no toxicological effects of the administration of StreptInCor in Wistar rats. The microscopic examination showed the presence of chronic granulomatous inflammation at the site of the StreptInCor and placebo injections (skeletal muscle) due to the presence of the adjuvant (aluminum hydroxide) in the formulations (33).

Adjuvants are important for enhancing the specific immune response against antigens in vaccines. Aluminum hydroxide induces the migration of neutrophils and eosinophils as well as dendritic cells and monocytes/macrophages to the site of inoculation (34, 35). Eosinophils are able to secrete preformed IL-4 and may play an immunomodulatory role in the activation of B cells elicited by aluminum hydroxide (36). Dendritic cells and macrophages function as inducers of adaptive immunity by presenting antigens to T cells (33, 37).

A major concern with the development of an M protein-based GAS vaccine is the possibility of inducing an autoimmune response, such as RF/RHD. Antibodies raised against the N-terminal region have been linked to RF/RHD pathogenesis (4, 18, 38). The C-terminal region that is conserved did not induce cross-reactive antibodies. In this context, in previous studies on isogenic, outbred, and HLA class II-transgenic mice immunized with StreptInCor (24, 25), no heart-tissue cross-reactive antibodies or deleterious reactions in the heart (myocardium and valves) or other tissues were observed. The repeated dose toxicity test performed on Wistar rats presented here showed no signs of toxic reaction in the heart or any of the organs evaluated after the administration of the StreptInCor candidate vaccine.

## Conclusions

The results of repeated StreptInCor candidate injection in doses ranging from 50 to 200 μg/ml on Wistar rats presented here indicate that the StreptInCor candidate vaccine is non-toxic and well-tolerated in rodent animal models.

## Data Availability Statement

The raw data supporting the conclusions of this article will be made available by the authors, without undue reservation.

## Ethics Statement

The animal study was reviewed and approved by National Animal Experiment Control Council (CONCEA) and Committee for Animal Care and Use (COBEA) and international requirements based on the Guide for the Care and Use of Laboratory Animals.

## Author Contributions

LS-R, EP, and RA: performed animal experimentation and analysis of data. RS: contributed with manuscript preparation and analysis of results. LD: histopathology analysis and manuscript preparation. JK: director of Immunology lab. LG: analysis of the data and manuscript preparation. All authors contributed to the article and approved the submitted version.

## Conflict of Interest

The authors declare that the research was conducted in the absence of any commercial or financial relationships that could be construed as a potential conflict of interest.
